# Point-of-care testing, antibiotic prescribing, and prescribing confidence for respiratory tract infections in primary care: a prospective audit in 18 European countries

**DOI:** 10.3399/BJGPO.2021.0212

**Published:** 2022-04-06

**Authors:** Alike W van der Velden, Alma C van de Pol, Emily Bongard, Daniela Cianci, Rune Aabenhus, Anca Balan, Femke Böhmer, Valerija Bralić Lang, Pascale Bruno, Slawomir Chlabicz, Samuel Coenen, Annelies Colliers, Ana García-Sangenís, Hrachuhi Ghazaryan, Maciej Godycki-Ćwirko, Siri Jensen, Christos Lionis, Sanne R van der Linde, Lile Malania, Jozsef Pauer, Angela Tomacinschii, Akke Vellinga, Ihor Zastavnyy, Susanne Emmerich, Adam Zerda, Theo J Verheij, Herman Goossens, Christopher C Butler

**Affiliations:** 1 Julius Center for Health Sciences and Primary Care, University Medical Center Utrecht, Utrecht, The Netherlands; 2 Nuffield Department of Primary Care Health Sciences, University of Oxford, Oxford, UK; 3 Department of Public Health, University of Copenhagen, Copenhagen, Denmark; 4 Balan Medfam SRL, Cluj Napoca, Romania; 5 Institute of General Practice, Rostock University Medical Center, Rostock, Germany; 6 Department of Family Medicine, Andrija Stampar School of Public Health, School of Medicine, University of Zagreb, Zagreb, Croatia; 7 Département de Santé Publique, Centre Hospitalier Universitaire de Nice, Nice, France; 8 Department of Family Medicine, Medical University of Bialystok, Bialystok, Poland; 9 Department of Family Medicine and Population Health, University of Antwerp, Antwerp, Belgium; 10 Institut Universitari d'Investigació en Atenció Primària Jordi Gol, Barcelona, Spain; 11 Wigmore Clinic, Yerevan, Armenia; 12 Yerevan State Medical University, Yerevan, Armenia; 13 Centre for Family and Community Medicine, Faculty of Health Sciences, Medical University of Lodz, Lodz, Poland; 14 The Antibiotic Centre for Primary Care, Department of General Practice, University of Oslo, Oslo, Norway; 15 Clinic of Social and Family Medicine, School of Medicine, University of Crete, Crete, Greece; 16 National Center for Disease Control and Public Health, Tbilisi, Georgia; 17 Amer Science Management LLC, Tbilisi, Georgia; 18 DRC Drug Research Centre, Balatonfüred, Hungary; 19 University Clinic of Primary Medical Assistance, Nicolae Testemițanu State University of Medicine and Pharmacy, Chișinău, The Republic of Moldova; 20 School of Medicine, National University of Ireland, Galway, Ireland; 21 NGO Academy of Family Medicine of Ukraine, Lviv, Ukraine; 22 Abbott Rapid Diagnostics Germany GmbH, Cologne, Germany; 23 AMR Strategy & Development, Becton Dickinson and Co, North Carolina, NC, US; 24 Laboratory of Medical Microbiology, Vaccine & Infectious Disease Institute, University of Antwerp, Antwerp, Belgium

**Keywords:** primary health care, anti-bacterial agents, respiratory tract infections, diagnostics, C-reactive protein, group A streptococcus, GP, audit, confidence

## Abstract

**Background:**

Between-country differences have been described in antibiotic prescribing for respiratory tract infection (RTI) in primary care, but not yet for diagnostic testing procedures and prescribing confidence.

**Aim:**

To describe between-country differences in RTI management, particularly diagnostic testing and antibiotic prescribing, and investigate which factors relate to antibiotic prescribing and GPs’ prescribing confidence.

**Design & setting:**

Prospective audit in 18 European countries.

**Method:**

An audit of GP-registered patient, clinical, and management characteristics for patients presenting with sore throat and/or lower RTI (*n* = 4982), and GPs' confidence in their antibiotic prescribing decision. Factors related to antibiotic prescribing and confidence were analysed using multi-level logistic regression.

**Results:**

Antibiotic prescribing proportions varied considerably: <20% in four countries, and >40% in six countries. There was also considerable variation in point-of-care (POC) testing (0% in Croatia, Moldova, and Romania, and >65% in Denmark and Norway, mainly for C-reactive protein [CRP] and group A streptococcal [strep A] infection), and in laboratory or hospital-based testing (<3% in Hungary, the Netherlands, and Spain, and >30% in Croatia, Georgia, Greece, and Moldova, mainly chest X-ray and white blood cell counting). Antibiotic prescribing was related to illness severity, comorbidity, age, fever, and country, but not to having performed a POC test. In nearly 90% of consultations, GPs were confident in their antibiotic prescribing decision.

**Conclusion:**

Despite high confidence in decisions about antibiotic prescribing, there is considerable variation in the primary care of RTI in European countries, with GPs prescribing antibiotics overall more often than is considered appropriate. POC testing may enhance the quality of antibiotic prescribing decisions if it can safely reverse decisions confidently made on clinical grounds alone to prescribe antibiotics.

## How this fits in

A large between-country variation in antibiotic prescribing for RTI exists in European primary care. Despite huge variation in management variables, such as POC and laboratory or hospital-based diagnostic testing and antibiotic prescribing, GPs expressed high confidence in their antibiotic prescribing decisions. Antibiotic prescribing was attributed to patient-related factors and country, but not convincingly to POC testing. POC testing may enhance the quality of antibiotic prescribing if it can safely reverse decisions confidently made on clinical grounds alone.

## Introduction

Sequential studies by the European Centre for Disease Prevention and Control (ECDC) have identified important between-country differences in the numbers and class of antibiotics used,^1,2^ and there is evidence that this variation is not warranted on clinical grounds.^3^ Despite antibiotic surveillance and stewardship programmes in many countries, these differences persist.^4–7^ Prudent use of antibiotics is fundamental to contain antimicrobial resistance, reduce unnecessary exposure of patients to its side effects, contain costs, and guide patients expecting antibiotics for common infectious syndromic illness.^8–11^

About 80%–90% of antibiotics are prescribed in primary care, where overprescribing is common for RTI.^12,13^ Moreover, RTI is often of viral aetiology and self-limiting, making this condition the main target for improving the quality of antibiotic prescribing decisions.^14,15^ Challenges facing prescribers include uncertainty about aetiology, unavailability of POC diagnostic testing to aid prescribing decisions, unfamiliarity with current guidelines, risk-adverse prescribing behaviour, and non-evidence based patients’ expectations about effectiveness of antibiotics.^9,16–19^ Additional influences include healthcare system and cultural factors.^3,20,21^

POC testing for RTI management has been introduced in primary care thus far mainly to identify a strep A throat infection,^22^ and to use the biomarker CRP to inform antibiotic prescribing for acute cough.^23,24^ These POC tests have been shown to decrease antibiotic prescribing for patients with sore throat and acute cough in clinical trials.^22,25,26^ Uptake of POC testing into national guidelines and clinical practice varies, but the extent to which this explains between-country differences in antibiotic prescribing is unknown.

A point-prevalence audit survey (PPAS) was conducted of GPs’ management of patients presenting with symptoms of an RTI in 18 European countries. This enabled the authors to: 1) describe between-country differences in management of RTI, with focus on antibiotic prescribing and the use of POC and laboratory or hospital-based diagnostics; and 2) to investigate which factors relate to antibiotic prescribing and GPs’ confidence in their prescribing decisions.

## Method

This was a PPAS of patient, clinical, and management characteristics of patients presenting with symptoms of an RTI in 18 European countries that differed with respect to healthcare organisation, antibiotic use, and income level (high, upper, and lower-middle). Data on presentation and management, including confidence in their antibiotic prescribing decision, were anonymously registered by GPs. Patients were not informed, nor asked to provide informed consent as no personally identifiable information was collected. This procedure was approved by research ethics committees in all participating countries.

### Setting

The PPAS was performed in an established European Primary Care Research Network (https://www.value-dx.eu ). The registration period was January and February 2020, and each national team was asked to involve practices to enable delivering 200–250 registrations per country. As a formal power analysis was not considered needed for an audit, it was assumed that this number would provide enough information to describe management and between-country variation.

Practice size (number of GPs working in the practice) and the national teams’ preference to ask GPs to register a pre-defined number of consultations,or during a short period, or the complete period resulted in a different number of practices involved per country: Armenia (*n* = 5), Belgium (*n* = 6), Croatia (*n* = 6), Germany (*n* = 2), Denmark (*n* = 20), Spain (*n* = 6), France (*n* = 18), Georgia (*n* = 5), Greece (*n* = 6), Hungary (*n* = 5), Ireland (*n* = 6), Moldova (*n* = 4), the Netherlands (*n* = 12), Norway (*n* = 18), Poland (*n* = 8), Romania (*n* = 5), Ukraine (*n* = 4), and the UK (*n* = 7).

### Patients eligible for registration

GPs were instructed to sequentially register their consultations with patients of all ages with symptoms of either acute sore throat (duration <14 days) and/or acute cough (duration <28 days), and to not register patients with only nasal or ear symptoms.

### Data

Data were entered directly during the consultation in an online data capture system, Research Online, or entered later using paper Case Report Forms (see Supplementary Appendix S1). Data registration covered the following: patient characteristics (age and comorbidity: cardiovascular disease, chronic respiratory condition, diabetes, and other chronic condition); clinical presentation (fever, illness duration, and signs and symptoms); GPs’ measurements (temperature, respiratory rate, heart rate, oxygen saturation, and blood pressure); diagnostic testing (POC, laboratory or hospital-based, and chest X-ray in the facility or hospital); prescribing (antibiotic, antiviral, inhaled medication, and antihistamine); advice provided; and referral to hospital. At the end of the consultation, being aware of the result of the POC test but unaware of the result of laboratory or hospital-based testing, GPs rated their level of confidence in their decision whether or not to prescribe antibiotics using a 5-point Likert scale. Unknown and missing data, which was <1% for each variable, was regarded as ‘no’ or ‘not present’, and both immediate and delayed prescribing were considered as antibiotic prescribing.

### Analyses

Patient and management data are presented for the full sample as percentages with 95% confidence intervals (CIs) and ranges, and by country .

Factors related to antibiotic prescribing were analysed with mixed-effects logistic regression analysis where country, use of strep A and/or CRP POC testing, illness severity, age, presence of any comorbidity, fever, and duration of illness were included as fixed effects. Practice was included as random effect to take into account that patients were nested within practices. The same analysis was performed for two subgroups: 1) patients with diagnoses of a throat infection (pharyngitis, tonsillitis, laryngitis, and peritonsillar abscess) with strep A testing as fixed effect; and 2) patients with a lower respiratory tract diagnosis (bronchiolitis, acute bronchitis, pneumonia,wheezing, and exacerbation) with CRP testing as fixed effect.

As the outcome for confidence in the antibiotic prescribing decision indicated low frequencies for the options moderately certain and (very) uncertain it was decided to collapse these into ‘less certainty’, and compare with consultations where GPs indicated both degrees of certainty ([very] certain). Factors related to less certainty were analysed with mixed-effects logistic regression analysis where country, diagnostic testing (none, any POC, any laboratory or hospital, and POC plus laboratory or hospital), illness severity, age, presence of any comorbidity, fever, antibiotic prescribing, patient request for an antibiotic, suspected viral aetiology, and unclear aetiology were included as fixed effects, and practice as random effect. Results are presented as odds ratios (ORs) for each variable with 95% CIs. The statistical analyses were performed with IBM SPSS Statistics (version 26) and SAS Enterprise Guide (version 7.13).

## Results

A total of 4982 consultations of patients presenting with symptoms of an acute RTI were registered (from 211 in Norway to 355 in Armenia) between January and February 2020. Patients of all ages and with different comorbidities were registered. Their main symptoms, clinical assessments, severity rating, diagnoses, GPs’ confidence in their antibiotic prescribing decision, provided advice, and hospital referral are shown in Supplementary Table S1. Country variation in these variables is provided in Supplementary Table S2. Influenza-like illness, pharyngitis, and bronchitis were the most frequently used diagnostic categories. Only 3.2% of patients were classified as having severe illness and 2.3% were referred to hospital. Nearly 80% of patients received advice regarding symptomatic treatment and nearly 40% were advised or prescribed to take days off work or school.

### Diagnostic testing and medication prescribing for RTI

**Figure 1.**
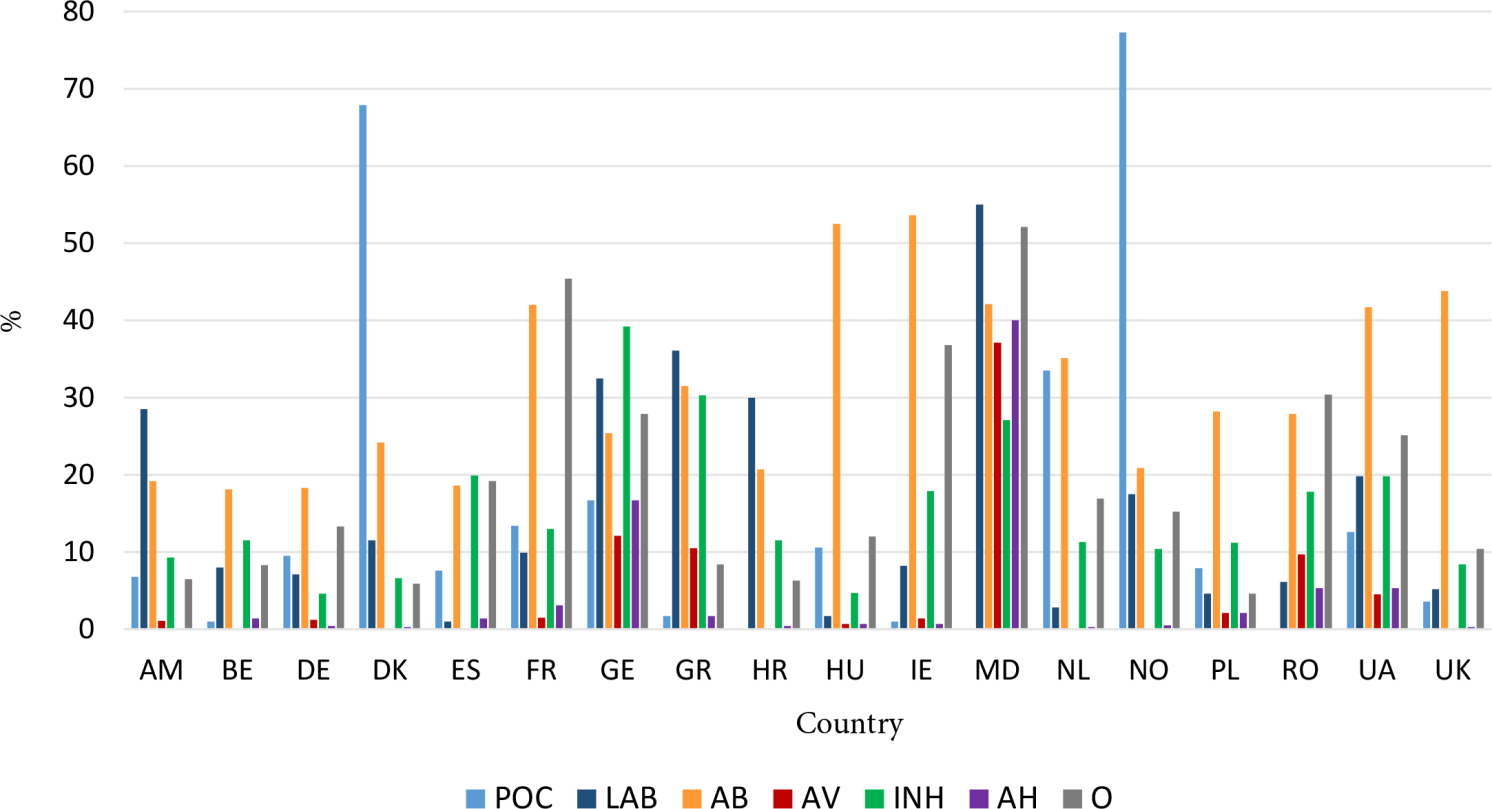
Country variation in point-of-care and laboratory or hospital-based diagnostic testing, and in medication prescribing for respiratory tract infection in primary care. AB = antibiotic. AH = antihistamine. AM = Armenia. AV = antiviral. BE = Belgium. DE = Germany. DK = Denmark. ES = Spain. FR = France. GE = Georgia. GR = Greece. HR = Croatia. HU = Hungary. IE = Ireland. INH = inhaled medication. LAB = laboratory or hospital-based. MD = Moldova. NL = the Netherlands. NO = Norway. O = other prescribed medication. PL = Poland. POC = point of care. UA = Ukraine. UK = United Kingdom.

Figure 1 shows between-country differences in diagnostic testing and medication prescribing for RTI. POC testing (mainly CRP and/or strep A) was common in Denmark, Norway, and the Netherlands, and virtually absent in Belgium, Greece, Croatia, Ireland, Moldova, and Romania. White blood cell differential counting was often done or requested in Norway, Ukraine, Greece, Croatia, and Moldova and hardly ever in Spain, Hungary, the Netherlands, Poland, Romania, and Ukraine. A chest X-ray was done at the facility or requested in the hospital for 7.2% of patients and for a high proportion of patients in Georgia (12.9% and 18.3%), Greece (1.7% and 29.8%), and Moldova (0% and 15.8%, Supplementary Table S2).

The proportion of patients prescribed antibiotics ranged from 18% (Belgium and Denmark) to >50% (Hungary and Ireland). Overall, 12.3% of patients requested an antibiotic prescription, ranging from <5% in Germany, Spain, and Croatia to >20% in Greece, Ireland, and Ukraine. Delayed antibiotic prescribing was highest in Belgium, Denmark, Ireland, and Ukraine. Broad-spectrum penicillins were most often prescribed (29.0%), followed by co-amoxicillin/clavulanate (22.2%, with high prescribing in Armenia, France, Georgia, Croatia, Moldova, Poland, Romania, and Ukraine) and macrolides (19.7%, with high prescribing in Georgia, Greece, Croatia, Poland, and Romania). Narrow-spectrum penicillins (overall 12.0%) were mainly prescribed in Denmark, Norway, and the UK. Cephalosporins (overall 7.5%) were more frequently prescribed in Hungary and Ukraine. Antivirals were frequently prescribed in Georgia, Greece, Moldova, and Romania (Figure 1 and Supplementary Table S2).

### Factors associated with antibiotic prescribing

Established prognostic factors, such as illness severity, older age, presence of comorbidity, fever, and longer illness duration, were all associated with antibiotic prescribing (Table 1). In the full sample, having performed POC testing was not associated with antibiotic prescribing. Country was associated with antibiotic prescribing also when adjusted for aforementioned prognostic factors (see Supplementary Table S3), meaning that the differences in prescribing proportions between countries are not owing to country variation in case mix and POC testing.

**Table 1.** Multivariable analysis of factors associated with antibiotic prescribing

Category	All (strep A and CRP)		Lower RTI (CRP)		Sore throat (strep A)	
	Adj OR	95% CI	Adj OR	95% CI	Adj OR	95% CI
Use of POC test(s)	1.3	0.9 to 1.8	0.7	0.4 to 1.1	**2.4**	**1.3 to 4.3**
Moderate illness severity	**7.4**	**6.2 to 8.9**	**5.3**	**3.8 to 7.3**	**9.5**	**6.9 to 13.0**
Severe illness	**8.0**	**5.4 to 12.0**	**4.1**	**2.3 to 7.2**	**3.6**	**1.2 to 11.0**
Age, year	**1.02**	**1.01 to 1.02**	**1.02**	**1.02 to 1.03**	**1.01**	**1.00 to 1.02**
Comorbidity	**1.3**	**1.**1 **to 1.5**	1.1	0.8 to 1.5	1.0	0.6 to 1.4
Fever	**1.6**	**1.3 to 1.9**	**2.9**	**2.1 to 4.0**	**2.0**	**1.5 to 2.8**
Duration (day)	**1.03**	**1.02 to 1.04**	**1.03**	**1.00 to 1.05**	**1.05**	**1.00 to 1.10**
Country	OR for 17 countries shown in Supplementary Table S3					

Multivariable mixed-effects logistic regression for the dependent variable antibiotic prescribing controlling for country, use of group A streptococcal (strep A) and/or C-reactive protein (CRP) point-of-care (POC) testing for all patients, CRP testing for patients with lower respiratory tract infection, and strep A testing for patients with sore throat, illness severity, age, comorbidity, fever, and illness duration. Practice was included as random effect. Reference categories are Belgium (lowest antibiotic prescribing proportion) for country; ‘no’ for POC testing, comorbidity, and fever; ‘mild’ for severity; and for age and duration an increase of one unit is considered. Bold indicates significance. OR = odds ratio. RTI = respiratory tract infection.

When analysed by subgroup, antibiotics were less often prescribed for patients with lower RTI diagnoses when CRP POC testing was done (OR 0.67, 95% CI = 0.39 to 1.1, statistically not significant in this sample), while more often for patients with sore throat infection diagnoses when a strep A POC test was done (OR 2.4, 95% CI = 1.3 to 4.3).

### Confidence in the antibiotic prescribing decision

**Figure 2.**
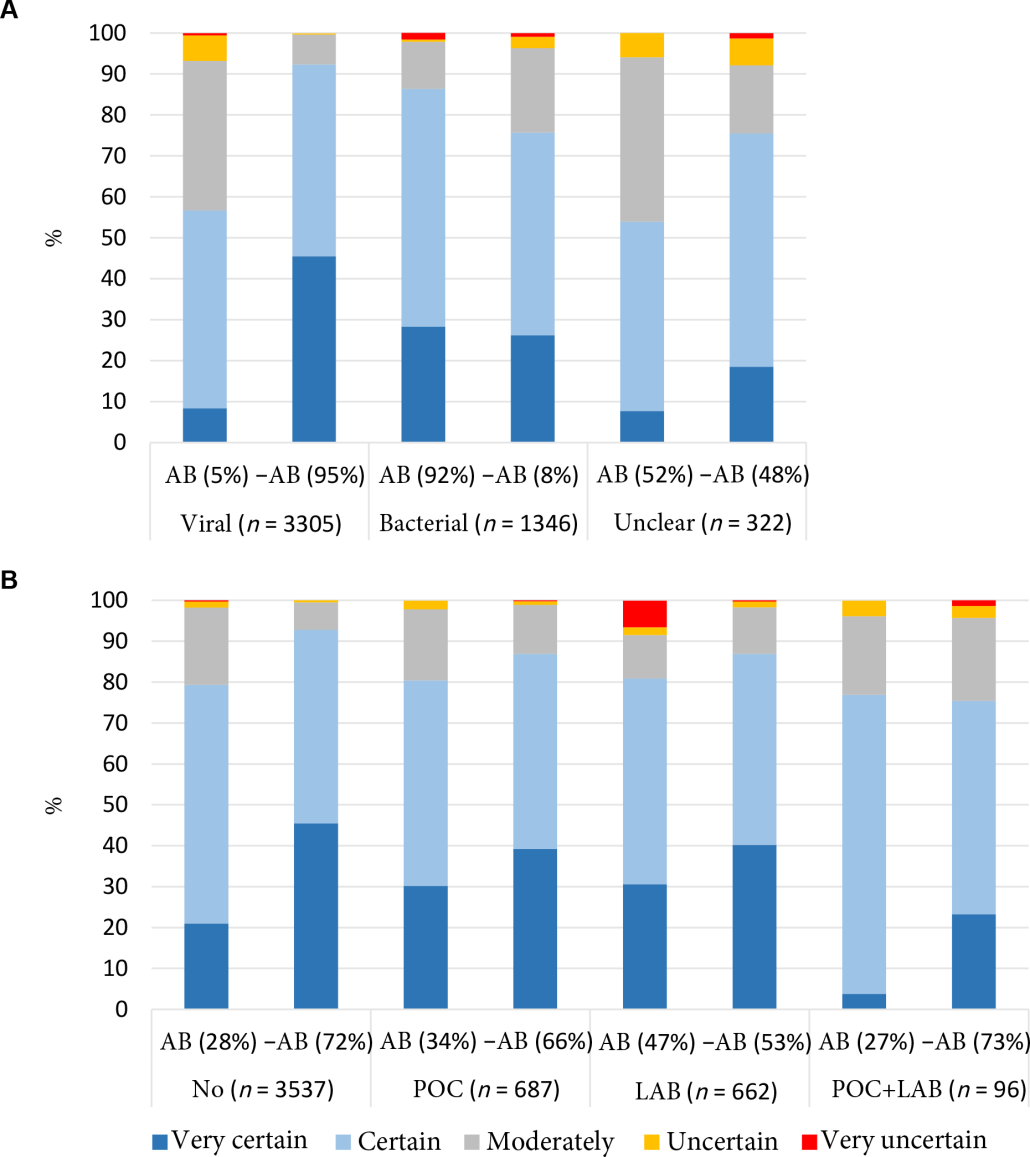
Percentages of consultations by level of confidence in the antibiotic prescribing decision; A) split by suspected viral, bacterial, and unclear aetiology; and B) split by testing group. AB = antibiotic prescribed. –AB = no antibiotic prescribed. POC = point-of-care. LAB = laboratory or hospital-based. POC+LAB = point-of-care plus laboratory or hospital-based.

GPs were generally confident in their antibiotic prescribing decision; in 88% of consultations, they rated their level of confidence as (very) certain (Supplementary Table S1). Unadjusted data show that GPs expressed lower confidence when they prescribed an antibiotic for patients with a suspected viral or an unclear aetiology, both relatively small subgroups (Figure 2A). Confidence when prescribing an antibiotic was not higher at the end of the consultation when POC testing was used, and not lower when laboratory or hospital-based testing was requested (Figure 2B). Confidence when no antibiotic was prescribed was somewhat lower when POC and/or laboratory testing was used or requested. After adjusting for multiple variables, including country, less certainty was associated with moderate or severe illness (OR 1.9, 95% CI = 1.5 to 2.4), POC testing (OR 1.5, 95% CI = 1.1 to 2.1), antibiotic prescribing (OR 3.3, 95% CI = 2.4 to 4.5), suspected viral aetiology (OR 2.4, 95% CI = 1.7 to 3.4), and unclear aetiology (OR 7.1, 95% CI = 5.0 to 10.o) (data not shown). Supplementary Figure S1, displaying confidence per diagnosis, shows high confidence in the prescribing decision for pharyngitis and tonsillitis, and lower confidence for influenza-like illness and bronchitis.

## Discussion

### Summary

This prospective 18-country audit of nearly 5000 primary care consultations for RTI confirms considerable variation in both the proportion and the class of antibiotics prescribed.^6,7^ There was also considerable variation in clinical assessments, POC, and laboratory or hospital-based testing, and in non-antibiotic prescriptions. However, GPs consistently expressed a high level of confidence in their decision whether or not to prescribe antibiotics. Antibiotic prescribing differences were explained most by country-level factors and individual illness severity, rather than whether or not POC testing was used.

### Strengths and limitations

The strength of this study is the uniform, prospective data capture, implemented in many contrasting European countries, including some that have not previously been included in prospective international studies of RTI in primary care. The PPAS provides insight in various management variables and enables between-country comparisons. The anonymous, audit-type procedure of registering sequentially presenting patients avoided selection bias as to whom to register or not. Therefore, it is considered that the study population is a representative sample of patients presenting with RTI symptoms in the participating countries, without enrichment for patients with certain characteristics or management variables. Filling out the CRF could easily be done during or immediately after the consultation without practical constraints of informing patients and taking informed consent. Owing to this minimal study burden on GPs, the PPAS can easily and usefully be repeated to capture changes in care provision, which was done at the start of the COVID-19 pandemic.^27^

Several weaknesses need to be considered. First, with respect to the generalisability of the results that have been presented at country level. Given the limited number of general practices participating in each country, outcomes might not represent primary care provided in the country. For instance, in Moldova, four practices in a university-linked primary care clinic participated, and these practices may have therefore been atypical. Second, concerning POC testing, heterogeneous data were dealt with. In some practices and/or countries, POC testing was not available, in some, GPs could choose whether or not to use the testing, while in others it was part of the guidelines. Whether POC testing was at the discretion of the GP, or not, the PPAS did provide an extensive dataset of consultations with and without POC testing and antibiotic prescribing. This allowed for comparing consultations with and without POC testing. This group-wise comparison indicated that overall confidence was not higher when POC had been used. However, reasons for deciding whether or not to perform a test and confidence before testing were not captured. Such information could have added to the understanding of the influence of performing a POC test on antibiotic prescribing and on changing confidence within the consultation. A Swedish study, for instance, indicated the influence of CRP testing on GPs’ degree of suspicion of pneumonia.^28^ Finally, the audit did not follow-up patients, so the clinical outcomes and hospital or laboratory-based test results were unknown. These data could have been helpful to determine whether GPs’ prescribing confidence was justified.

### Comparison with existing literature

Several other studies have described the differences in numbers, types, and appropriateness of prescribed antibiotics between European countries.^1,3,6,7,19,29,30^ The prescribing proportion in the PPAS were not always congruent with the ranking in ECDC reports^6,7^ in which, for example, the Netherlands ranks lowest and Belgium considerably higher. However, overall antibiotic use is not only determined by the prescribing proportion, but also by the number of patients that yearly present to a GP, the denominator. In countries with a high threshold to consult the GP, fewer patients overall, but probably with relatively more severe illness, will present, as opposed to, for example, a country where all patients with RTI consult their GP for a sick note. The PPAS captured data from a pre-defined number of consultations per country and not on total numbers of consulting patients in the 2 months‘ registration period.

Variation in the use of POC testing for RTI has been described for practices and GPs within some countries,^31^ but not in a prospective multi-country study using the same data capture form.

Randomised controlled trials have found that strep A and CRP POC testing reduce antibiotic prescribing at the index consultation.^4,22,25,26,32^ However, pragmatic studies on the use of POC testing in general practice show smaller, often statistically non-significant effects.^33^ The routine care data captured in the audit pointed towards a different effect of CRP and strep A testing. CRP testing tended to decrease antibiotic prescribing and the non-significance might have been owing to using routine care data and/or the sample size. Strep A testing on the other hand was associated with higher antibiotic prescribing; testing might have been used to confirm an antibiotic prescribing decision. Although the PPAS was not designed to specifically investigate factors influencing antibiotic prescription, the results indicate that trial results need to be confirmed with data from routine primary care practice.

### Implications for practice

Countries where POC testing is widely used, including Denmark, Norway, Sweden, and the Netherlands, have lower antibiotic use compared with countries where POC testing is not widely implemented.^34^ However, this association is not necessarily causal. The PPAS highlighted a strong ‘country factor’ associated with antibiotic prescribing. This country factor is no doubt the product of many interacting factors, such as antibiotic prescribing levels before the implementation of POC testing, national prescribing guidelines, antimicrobial stewardship programmes, and culturally determined GP and patient-related factors.

GPs have high confidence in their antibiotic prescribing decisions, including when POC testing was not used or available. It can be questioned whether the confidently made yes-antibiotic prescribing decisions are correct, given that antibiotics were prescribed in 70% of patients with tonsillitis, 45% of patients with bronchitis, and 19% of patients with pharyngitis, which is higher than considered appropriate.^13^ In implementing POC testing in guidelines and primary care practice, this expressed confidence would support the advice to use POC testing when the GP is in doubt to increase their confidence in diagnosis and/or prescribing. Additionally, POC testing for RTI might have more impact as a standard antibiotic stewardship intervention implemented to safely deviate from a (confidently made) yes-antibiotic prescribing decision.

In conclusion, despite high confidence of GPs in their decisions about antibiotic prescribing, there is considerable variation in primary care management of RTIs in European countries, with many GPs prescribing antibiotics more often than considered appropriate. POC testing may enhance antibiotic prescribing stewardship even in the face of GPs being confident in prescribing, if it can safely reverse an antibiotic prescribing decision made on clinical grounds alone. The PPAS is considered a valuable research tool and its country-specific information can aid in designing and implementing interventions for RTI management taking the country’s specific context into account.
